# Crystal structure and Hirshfeld surface analysis and energy frameworks of 1-(2,4-di­methyl­phen­yl)-4-(4-meth­oxy­phen­yl)naphthalene

**DOI:** 10.1107/S2056989018008332

**Published:** 2018-06-08

**Authors:** U. Mohamooda Sumaya, J. KarunaKaran, K. Biruntha, A. K. MohanaKrishnan, G. Usha

**Affiliations:** a Department of Physics, Bharathi Women’s College (A), Chennai-108, Tamilnadu, India; b Department of Organic Chemistry, University of Madras, Chennai-25, Tamilnadu, India; c PG and Research Department of Physics, Queen Mary’s College (A), Chennai-4, Tamilnadu, India

**Keywords:** crystal structure, naphthalene, C—H⋯π inter­actions, Hirshfeld surface analysis, two-dimensional fingerprint plots, energy frameworks.

## Abstract

In the title compound, C_25_H_22_O, the two rings of the naphthalene system are inclined to each other by 3.06 (15)°. The mean plane of the naphthalene ring system makes a dihedral angle of 65.24 (12)° with the di­methyl­phenyl ring and 55.82 (12)° with the meth­oxy­phenyl ring. The di­methyl­phenyl ring is inclined to the meth­oxy­phenyl ring by 59.28 (14)°. In the crystal, adjacent mol­ecules are linked *via* C—H⋯π inter­actions, forming chains along [100]. Using Hirshfeld surface and two-dimensional fingerprint plots, the presence of short inter­molecular inter­actions in the crystal structure were analysed. The inter­molecular inter­action energies were also calculated and their distribution over the crystal structure was visualized graphically using energy frameworks.

## Abstract 2

In the title naphthalene derivative, the mean plane of the naphthalene ring system makes dihedral angles of 65.24 (12)° with the di­methyl­phenyl ring and 55.82 (12)° with meth­oxy­phenyl ring. The latter two rings are inclined to each other by 59.28 (14)°.

## Chemical context   

Naphthalene and its derivatives are known for their wide range of applications in the field of pharmaceuticals. They are also used in the manufacturing of colorants, surface-active agents, resins, disinfectants and insecticides. These derivatives play a vital role in the control of microbial infection (Rokade & Sayyed, 2009 ▸) and in the chemical defence against biological enemies (Wright *et al.*, 2000 ▸). Compounds with a naphthalene moiety have been shown to exhibit significant anti-TB activity (Upadhayaya *et al.*, 2010 ▸).
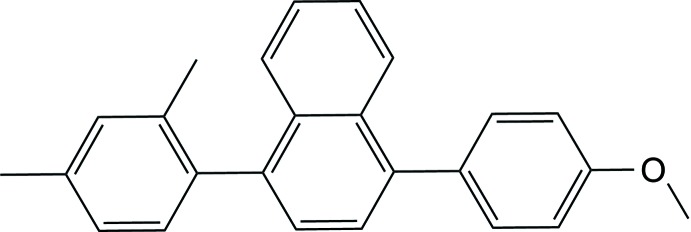


## Structural commentary   

**Figure 1:**
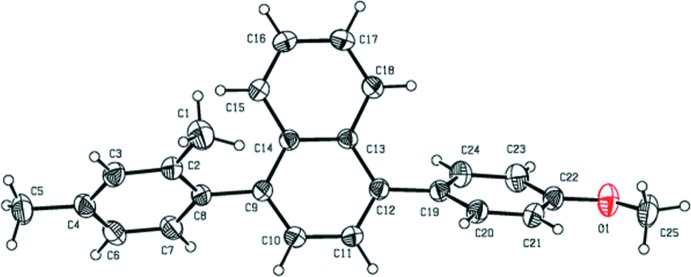
The mol­ecular structure of the title compound, with the atom labelling. Displacement ellipsoids are drawn at 50% probability level.

The mol­ecular structure of the title compound is illustrated in Fig. 1 ▸. The benzene ring (C9–C14) of the naphthalene moiety is substituted by a di­methyl­phenyl ring (C2–C4/C6–C8) and a meth­oxy­phenyl ring (C19–C24) *para* to each other. The naphthalene ring system is slightly bent with the two aryl rings being inclined to each other by 3.06 (15)°. Its mean plane makes dihedral angles of 65.24 (12)° with the di­methyl­phenyl ring (C2–C4/C6–C8) and 55.82 (12)° with meth­oxy­phenyl ring (C19–C24). The latter two rings are inclined to each other by 59.28 (14)°. The meth­oxy group (C22/O1/C25) lies out of the plane of the benzene ring (C19–C24) to which it is attached by 11.3 (3)°. The bond lengths and bond angles are similar to those reported for 1,4-di­phenyl­naphthalene, which crystallized with two independent mol­ecules in the asymmetric unit (Lima *et al.*, 2012 ▸).

## Supra­molecular features   

**Figure 2:**
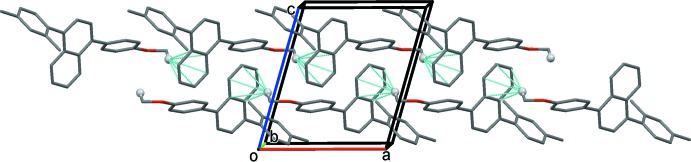
The crystal packing of the title compound, viewed along the *b* axis. The C—H⋯π inter­actions (see Table 1 ▸) are shown as dashed lines, and only the H atom H25*C* (grey ball) has been included.

In the crystal, there is only one significant inter­molecular inter­action present, *viz*. a C—H⋯π inter­action linking adjacent mol­ecules to form chains propagating along the *a*-axis direction (Table 1 ▸ and Fig. 2 ▸).

## Database survey   

A search of the Cambridge Structural Database (Version 5.39, last update February 2018; Groom *et al.*, 2016 ▸) for the aromatic skeleton of the title compound yielded ten hits. They include 1,4-di­phenyl­naphthalene itself, which crystallized with two independent mol­ecules in the asymmetric unit (CSD refcode ZAXJEP: Lima *et al.*, 2012 ▸). There are also a number of copper(II) complexes (LAYQOU: Chen *et al.*, 2017 ▸; BOSHIC: Cai *et al.*, 2014 ▸; PUBSOV: Lin *et al.*, 2009 ▸) of the tetra­carb­oxy­lic acid derivative, 5,5′-(naphthalene-1,4-di­yl)diisophthalic acid, all of which are metal–organic frameworks.

## Analysis of the Hirshfeld surfaces, inter­action energies and energy frameworks   

**Figure 3:**
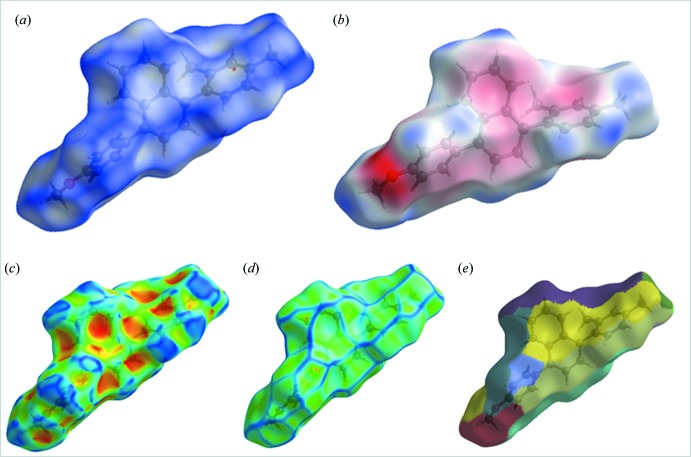
Hirshfeld surfaces mapped over (*a*) *d*_norm_, (*b*) electrostatic potential, (*c*) shape index and (*d*) curvedness and (*e*) fragment patches.

**Figure 4:**
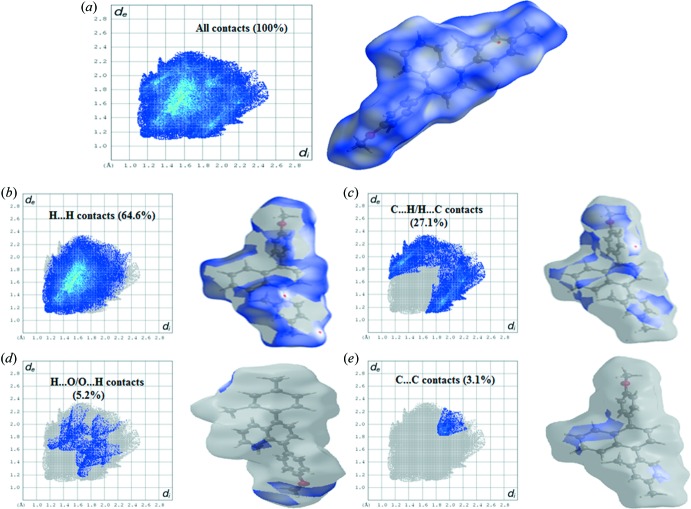
Two-dimensional fingerprint plot for the title compound showing the contributions of individual types of inter­actions: (*a*) all inter­molecular contacts, (*b*) H⋯H contacts, (*c*) C⋯H/H⋯C contacts, (*d*) H⋯O/O⋯H contacts, (*e*) C⋯C contacts. The outline of the full fingerprint is shown in grey. Surfaces to the right highlight the relevant surface patches associated with the specific contacts with *d*_norm_ mapped.

The Hirshfeld surfaces and two-dimensional fingerprint plots were generated in order to explore and qu­antify the weak inter­molecular inter­actions using the program *CrystalExplorer* 17.5 (Turner *et al.*, 2017 ▸). The electrostatic potentials were calculated using *TONTO*, integrated in the program *CrystalExplorer* (Spackman *et al.*, 2008 ▸; Jayatilaka *et al.*, 2005 ▸). The Hirshfeld surfaces of the title compound were mapped over *d*_norm_, electrostatic potential, curvedness and shape index (Fig. 3 ▸ *a*–3*d*); depending upon the closeness to the adjacent mol­ecules, the colour patches are mapped differently on the Hirshfeld surface (Fig. 3 ▸ *e*). Two-dimensional fingerprint plots showing the result of all inter­molecular contacts (McKinnon *et al.*, 2007 ▸) are presented in Fig. 4 ▸ *a*; *d*_i_ (*x* axis) and *d*_e_ (*y* axis) are the closest inter­nal and external distance from a given point on the Hirshfeld surface. The fingerprint plot of H⋯H contacts, which represent the largest contribution to the Hirshfeld surface (64.6%), are shown as a distinct pattern with a minimum value of *d*_e_ = *d*_i_ ≃ 1.2 Å (Fig. 4 ▸ *b*). The C⋯H/H⋯C inter­actions appear as the next largest region of the fingerprint plot, highly concentrated at the edges, having almost the same *d*_e_ + *d*_i_ ≃ 2.7 Å (Fig. 4 ▸ *c*), with on overall contribution of 27.1%. The O⋯H/H⋯O inter­actions on the fingerprint plot, which contribute 5.2% of the total Hirshfeld surfaces, with *d*_e_ + *d*_i_ ≃ 2.8 Å (Fig. 4 ▸ *d*) are shown as two symmetrical wings. The C⋯C contacts, which are the measure of π–π stacking inter­actions, occupy 3.1% of the Hirshfeld surfaces and appear as a unique triangle at about *d*_e_ = *d*_i_ ≃ 1.8 Å (Fig. 4 ▸ *e*). These weak inter­actions mostly contribute to the packing of the title compound.

**Figure 5:**
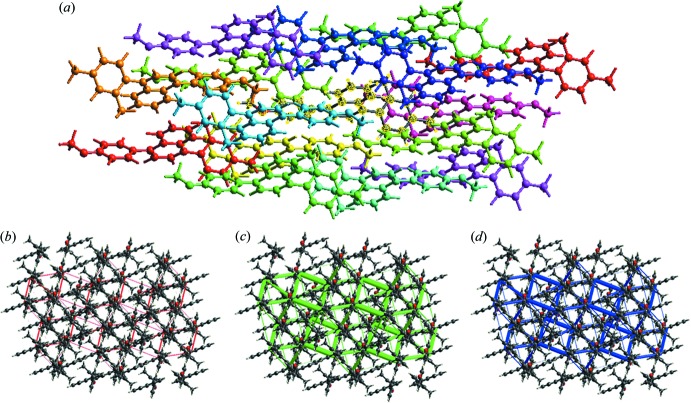
(*a*) Inter­action between the selected mol­ecule and the mol­ecules present in a 3.8 Å cluster around it, (*b*) Coulombic energy, (*c*) dispersion energy and (*d*) total energy.

The inter­action energies between the mol­ecules are obtained using monomer wavefunctions at the B3LYP/6-31G(p,d) level. The total inter­action energy, which is the sum of scaled components, was calculated for a 3.8 Å radius cluster of mol­ecules around the selected mol­ecule (Fig. 5 ▸ *a*). The scale factors used in the CE-B3LYP benchmarked energy model (Mackenzie *et al.*, 2017 ▸) are given in Table 2 ▸. The energies calculated by the energy model reveals that the dispersion energy contributes significantly to the inter­actions in the crystal (Table 3 ▸).

The energy framework calculations were performed for a cluster of mol­ecules present in 2 × 2 × 2 unit cells using the CE-B3LYP energy model. Energies between mol­ecular pairs are represented as cylinders joining the centroids of pairs of mol­ecules with the cylinder radius proportional to the magnitude of the inter­action energy. Energy frameworks were constructed for *E*_elec_ as red cylinders, *E*_dis_ as green and *E*_tot_ as blue (Fig. 5 ▸ *b*–5*d*) and these cylinders represent the relative strength of mol­ecular packing in different directions. Thus the supra­molecular architecture of the crystal structure is visualized uniquely by energy frameworks.

## Synthesis and crystallization   

**Figure 6:**
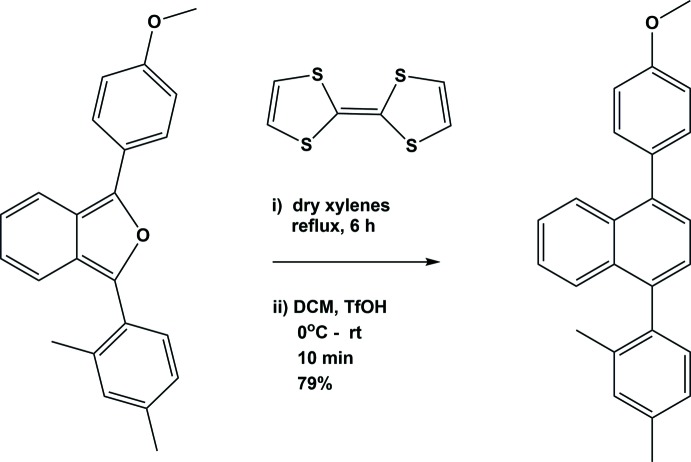
Reaction scheme.

A reaction scheme for the synthesis of the title compound is illustrated in Fig. 6 ▸. To a solution of *m*-xylyl-*p*-anisyl tethered benzo[*c*]furan (0.16 g, 0.49 mmol) in dry xylenes (15 ml) was added tetra­thia­fulvalene (TTF) (0.10 g, 0.49 mmol) and the mixture was refluxed until the consumption of benzo[*c*]furan was complete; monitored by the disappearance of the fluorescent colour after 6 h. After the removal of xylenes *in vacuo*, the crude adduct was dissolved in dry CH_2_Cl_2_ (15 ml) and then kept at 273 K. To this, triflic acid (0.02 g, 0.13 mmol) was added and the mixture stirred at room temperature for 10 min. After completion of the reaction (monitored by TLC), it was poured into ice–water (20 ml) and then extracted with CH_2_Cl_2_ (2 × 10 ml). The organic layers were combined and washed with aq. NaHCO_3_ (2 × 10 ml) and then dried (Na_2_SO_4_). Removal of the solvent followed by column chromatographic purification (silica gel, 5% ethyl acetate in hexa­ne) afforded the title compound as a yellow solid (0.20 g, 79%). Yellow block-like crystals of the title compound, suitable for X-ray diffraction analysis, were obtained by slow evaporation of a solution in CHCl_3_ (m.p. 351–353 K).

## Refinement   

Crystal data collection and structure refinement details are summarized in Table 4 ▸. All H atoms were positioned geometrically and refined using a riding model: C—H = 0.93–0.96 Å with *U*_iso_(H) = 1.5 *U*_eq_(C-meth­yl) and 1.2*U*_eq_(C) for other H atoms.

## Supplementary Material

Crystal structure: contains datablock(s) I, Global. DOI: 10.1107/S2056989018008332/su5445sup1.cif

Structure factors: contains datablock(s) I. DOI: 10.1107/S2056989018008332/su5445Isup2.hkl

Supporting information file. DOI: 10.1107/S2056989018008332/su5445Isup3.cml

CCDC reference: 1841351

Additional supporting information:  crystallographic information; 3D view; checkCIF report

## Figures and Tables

**Table 1:** Hydrogen-bond geometry (Å, °) *Cg* is the centroid of the C13–C18 ring.

*D*—H⋯*A*	*D*—H	H⋯*A*	*D*⋯*A*	*D*—H⋯*A*
C25—H25*C*⋯*Cg* ^i^	0.96	2.78	3.597 (5)	144

Symmetry code: (i) 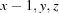.

**Table 2:** Scale factors for the benchmarked energy model

Energy model	*k*_elec_	*k*_pol_	*k*_energy-dispersive_	*k*_rep_
CE-B3LYP⋯B3LYP/6–31G(d,p) electron densities	1.057	0.740	0.871	0.618

**Table 3:** Inter­action energies (kJ mol^−1^) *R* is the distance between mol­ecular centroids (mean atomic position) in Å and *N* is the number of molecules at that distance.

Colour	*N*	symop	*R*	*E*_elec_	*E*_pol_	*E*_energy-dispersive_	*E*_rep_	*E*_total_
Red	2	*x*, *y*, *z*	15.38	−2.2	−0.6	−11.2	6.2	−8.6
Orange	1	−*x*, −*y*, −*z*	15.99	−4.3	−0.8	−11.5	4.2	−12.5
Yellow	1	−*x*, −*y*, −*z*	7.45	−6.2	−1.3	−39.2	19.3	−29.7
Lime	2	*x*, *y*, *z*	9.17	−10.0	−1.8	−44.0	26.8	−33.6
Green	2	*x*, *y*, *z*	10.46	−0.1	−0.1	−6.6	1.5	−5.0
Aqua­marine	1	−*x*, −*y*, −*z*	6.86	−6.8	−0.9	−39.9	18.7	−31.0
Cyan	1	−*x*, −*y*, −*z*	10.11	−0.3	−0.4	−19.8	6.7	−13.7
Blue	1	−*x*, −*y*, −*z*	5.37	−3.3	−1.9	−69.2	32.2	−45.2
Violet	1	−*x*, −*y*, −*z*	9.31	−6.5	−0.8	−36.0	20.7	−26.0
Orchid	2	*x*, *y*, *z*	14.01	0.1	0.0	−2.0	0.0	−1.7
Magenta	1	−*x*, −*y*, −*z*	11.61	−3.3	−1.0	−41.6	20.4	−27.9

**Table 4:** Experimental details

Crystal data	
Chemical formula	C_25_H_22_O
*M*_r_	338.42
Crystal system, space group	Triclinic, *P* 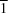
Temperature (K)	296
*a*, *b*, *c* (Å)	9.1670 (9), 10.4566 (10), 11.2499 (11)
α, β, γ (°)	64.707 (4), 71.312 (4), 77.032 (4)
*V* (Å^3^)	918.75 (16)
*Z*	2
Radiation type	Mo *K*α
μ (mm^−1^)	0.07
Crystal size (mm)	0.15 × 0.10 × 0.10

Data collection	
Diffractometer	Bruker Kappa APEXII CCD
Absorption correction	Multi-scan (*SADABS*; Bruker, 2012 ▸)
*T*_min_, *T*_max_	0.900, 0.945
No. of measured, independent and observed [*I* > 2σ(*I*)] reflections	19120, 3828, 1777
*R*_int_	0.060
(sin θ/λ)_max_ (Å^−1^)	0.631

Refinement	
*R*[*F* ^2^ > 2σ(*F* ^2^)], *wR*(*F* ^2^), *S*	0.057, 0.193, 1.00
No. of reflections	3828
No. of parameters	238
H-atom treatment	H-atom parameters constrained
Δρ_max_, Δρ_min_ (e Å^−3^)	0.27, −0.19

Computer programs: *APEX2*, *SAINT* and *XPREP* (Bruker, 2012 ▸), *SHELXT2014* (Sheldrick, 2015*a* ▸), *SHELXL2014* (Sheldrick, 2015*b* ▸), *ORTEP-3 for Windows* (Farrugia, 2012 ▸) and *Mercury* (Macrae *et al.*, 2008 ▸).

## supplementary crystallographic information

### Crystal data

**Table:**

C_25_H_22_O	*Z* = 2
*M**_r_* = 338.42	*F*(000) = 360
Triclinic, *P*1	*D*_x_ = 1.223 Mg m^−^^3^
*a* = 9.1670 (9) Å	Mo *K*α radiation, λ = 0.71073 Å
*b* = 10.4566 (10) Å	Cell parameters from 19154 reflections
*c* = 11.2499 (11) Å	θ = 2.3–22.7°
α = 64.707 (4)°	µ = 0.07 mm^−^^1^
β = 71.312 (4)°	*T* = 296 K
γ = 77.032 (4)°	Block, yellow
*V* = 918.75 (16) Å^3^	0.15 × 0.10 × 0.10 mm

### Data collection

**Table:**

Bruker Kappa APEXII CCD diffractometer	1777 reflections with *I* > 2σ(*I*)
Radiation source: fine-focus sealed tube	*R*_int_ = 0.060
ω and φ scan	θ_max_ = 26.6°, θ_min_ = 2.1°
Absorption correction: multi-scan (SADABS; Bruker, 2012)	*h* = −11→11
*T*_min_ = 0.900, *T*_max_ = 0.945	*k* = −13→13
19120 measured reflections	*l* = −14→14
3828 independent reflections	

### Refinement

**Table:**

Refinement on *F*^2^	Primary atom site location: structure-invariant direct methods
Least-squares matrix: full	Secondary atom site location: difference Fourier map
*R*[*F*^2^ > 2σ(*F*^2^)] = 0.057	Hydrogen site location: inferred from neighbouring sites
*wR*(*F*^2^) = 0.193	H-atom parameters constrained
*S* = 1.00	*w* = 1/[σ^2^(*F*_o_^2^) + (0.0922*P*)^2^ + 0.0768*P*] where *P* = (*F*_o_^2^ + 2*F*_c_^2^)/3
3828 reflections	(Δ/σ)_max_ < 0.001
238 parameters	Δρ_max_ = 0.27 e Å^−^^3^
0 restraints	Δρ_min_ = −0.19 e Å^−^^3^

### Special details

**Table:**

Geometry. All esds (except the esd in the dihedral angle between two l.s. planes) are estimated using the full covariance matrix. The cell esds are taken into account individually in the estimation of esds in distances, angles and torsion angles; correlations between esds in cell parameters are only used when they are defined by crystal symmetry. An approximate (isotropic) treatment of cell esds is used for estimating esds involving l.s. planes.

### Fractional atomic coordinates and isotropic or equivalent isotropic displacement parameters (Å^2^)

**Table:**

	*x*	*y*	*z*	*U*_iso_*/*U*_eq_	
C1	0.7907 (4)	0.8345 (4)	0.2984 (3)	0.0771 (10)	
H1A	0.7017	0.8047	0.2937	0.116*	
H1B	0.7750	0.9350	0.2771	0.116*	
H1C	0.8053	0.7849	0.3885	0.116*	
C2	0.9307 (3)	0.8015 (3)	0.1987 (3)	0.0509 (7)	
C3	1.0610 (3)	0.8748 (3)	0.1542 (3)	0.0578 (8)	
H3	1.0583	0.9411	0.1898	0.069*	
C4	1.1927 (3)	0.8530 (3)	0.0602 (3)	0.0573 (8)	
C5	1.3309 (4)	0.9324 (4)	0.0196 (4)	0.0863 (11)	
H5A	1.4237	0.8679	0.0169	0.129*	
H5B	1.3205	0.9724	0.0848	0.129*	
H5C	1.3365	1.0071	−0.0686	0.129*	
C6	1.1935 (3)	0.7571 (3)	0.0065 (3)	0.0598 (8)	
H6	1.2803	0.7415	−0.0585	0.072*	
C7	1.0668 (3)	0.6835 (3)	0.0480 (3)	0.0541 (7)	
H7	1.0699	0.6198	0.0093	0.065*	
C8	0.9353 (3)	0.7015 (3)	0.1453 (3)	0.0446 (7)	
C9	0.8049 (3)	0.6158 (3)	0.1869 (3)	0.0437 (6)	
C10	0.7284 (3)	0.6328 (3)	0.0942 (3)	0.0537 (7)	
H10	0.7534	0.7037	0.0079	0.064*	
C11	0.6138 (3)	0.5472 (3)	0.1247 (3)	0.0527 (7)	
H11	0.5648	0.5633	0.0581	0.063*	
C12	0.5716 (3)	0.4408 (3)	0.2488 (3)	0.0426 (6)	
C13	0.6437 (3)	0.4220 (3)	0.3514 (2)	0.0393 (6)	
C14	0.7621 (3)	0.5075 (3)	0.3195 (2)	0.0403 (6)	
C15	0.8360 (3)	0.4807 (3)	0.4219 (3)	0.0507 (7)	
H15	0.9173	0.5323	0.4017	0.061*	
C16	0.7907 (3)	0.3810 (3)	0.5493 (3)	0.0602 (8)	
H16	0.8414	0.3649	0.6147	0.072*	
C17	0.6690 (3)	0.3029 (3)	0.5823 (3)	0.0612 (8)	
H17	0.6361	0.2374	0.6706	0.073*	
C18	0.5983 (3)	0.3221 (3)	0.4860 (3)	0.0518 (7)	
H18	0.5180	0.2682	0.5092	0.062*	
C19	0.4540 (3)	0.3483 (3)	0.2743 (2)	0.0432 (6)	
C20	0.3108 (3)	0.4064 (3)	0.2491 (3)	0.0504 (7)	
H20	0.2877	0.5046	0.2182	0.060*	
C21	0.2007 (3)	0.3235 (3)	0.2682 (3)	0.0525 (7)	
H21	0.1051	0.3658	0.2507	0.063*	
C22	0.2331 (3)	0.1787 (3)	0.3130 (3)	0.0510 (7)	
C23	0.3750 (3)	0.1177 (3)	0.3379 (3)	0.0601 (8)	
H23	0.3977	0.0195	0.3676	0.072*	
C24	0.4841 (3)	0.2011 (3)	0.3193 (3)	0.0543 (7)	
H24	0.5794	0.1581	0.3371	0.065*	
C25	−0.0236 (4)	0.1430 (4)	0.3343 (4)	0.0834 (11)	
H25A	−0.0854	0.0667	0.3644	0.125*	
H25B	−0.0284	0.2070	0.2437	0.125*	
H25C	−0.0624	0.1932	0.3942	0.125*	
O1	0.1324 (2)	0.0867 (2)	0.3352 (2)	0.0742 (7)	

### Atomic displacement parameters (Å^2^)

**Table:**

	*U*^11^	*U*^22^	*U*^33^	*U*^12^	*U*^13^	*U*^23^
C1	0.076 (2)	0.079 (2)	0.081 (2)	−0.0173 (19)	−0.0011 (18)	−0.044 (2)
C2	0.0503 (17)	0.0521 (17)	0.0505 (17)	−0.0106 (14)	−0.0132 (14)	−0.0171 (14)
C3	0.068 (2)	0.0485 (17)	0.0622 (19)	−0.0158 (15)	−0.0235 (16)	−0.0169 (15)
C4	0.0534 (18)	0.0526 (18)	0.0532 (18)	−0.0149 (15)	−0.0173 (15)	−0.0013 (15)
C5	0.069 (2)	0.091 (3)	0.088 (3)	−0.040 (2)	−0.0228 (19)	−0.007 (2)
C6	0.0523 (18)	0.0585 (19)	0.0533 (18)	−0.0127 (15)	−0.0040 (14)	−0.0106 (16)
C7	0.0547 (17)	0.0528 (18)	0.0498 (17)	−0.0128 (14)	−0.0061 (14)	−0.0169 (14)
C8	0.0491 (16)	0.0419 (15)	0.0427 (15)	−0.0124 (13)	−0.0133 (13)	−0.0113 (13)
C9	0.0458 (15)	0.0475 (16)	0.0415 (15)	−0.0104 (13)	−0.0101 (12)	−0.0186 (13)
C10	0.0621 (18)	0.0550 (18)	0.0421 (16)	−0.0192 (15)	−0.0150 (14)	−0.0093 (14)
C11	0.0593 (18)	0.0571 (18)	0.0424 (17)	−0.0159 (15)	−0.0180 (14)	−0.0113 (14)
C12	0.0412 (15)	0.0454 (15)	0.0438 (16)	−0.0070 (12)	−0.0088 (12)	−0.0197 (13)
C13	0.0393 (14)	0.0416 (15)	0.0365 (15)	−0.0044 (12)	−0.0067 (11)	−0.0165 (12)
C14	0.0404 (14)	0.0416 (15)	0.0408 (15)	−0.0055 (12)	−0.0091 (12)	−0.0178 (13)
C15	0.0515 (16)	0.0574 (18)	0.0482 (17)	−0.0131 (14)	−0.0149 (14)	−0.0197 (15)
C16	0.069 (2)	0.070 (2)	0.0467 (18)	−0.0158 (17)	−0.0230 (15)	−0.0174 (16)
C17	0.073 (2)	0.067 (2)	0.0392 (17)	−0.0215 (17)	−0.0110 (15)	−0.0115 (15)
C18	0.0526 (17)	0.0563 (18)	0.0454 (17)	−0.0149 (14)	−0.0063 (13)	−0.0185 (15)
C19	0.0435 (15)	0.0456 (16)	0.0436 (15)	−0.0065 (13)	−0.0088 (12)	−0.0206 (13)
C20	0.0522 (17)	0.0465 (16)	0.0551 (17)	−0.0068 (14)	−0.0183 (13)	−0.0179 (14)
C21	0.0458 (16)	0.0576 (19)	0.0610 (18)	−0.0057 (14)	−0.0174 (13)	−0.0261 (15)
C22	0.0521 (18)	0.0548 (19)	0.0544 (18)	−0.0148 (15)	−0.0075 (14)	−0.0285 (15)
C23	0.060 (2)	0.0463 (17)	0.076 (2)	−0.0056 (16)	−0.0136 (16)	−0.0280 (16)
C24	0.0451 (16)	0.0518 (18)	0.0666 (19)	−0.0019 (14)	−0.0130 (14)	−0.0256 (15)
C25	0.060 (2)	0.102 (3)	0.108 (3)	−0.029 (2)	−0.0172 (19)	−0.051 (2)
O1	0.0641 (14)	0.0687 (14)	0.1023 (18)	−0.0233 (12)	−0.0140 (12)	−0.0415 (13)

### Geometric parameters (Å, º)

**Table:**

C1—C2	1.495 (4)	C13—C18	1.413 (3)
C1—H1A	0.9600	C13—C14	1.421 (3)
C1—H1B	0.9600	C14—C15	1.416 (3)
C1—H1C	0.9600	C15—C16	1.360 (4)
C2—C8	1.399 (4)	C15—H15	0.9300
C2—C3	1.401 (3)	C16—C17	1.390 (3)
C3—C4	1.375 (4)	C16—H16	0.9300
C3—H3	0.9300	C17—C18	1.357 (4)
C4—C6	1.373 (4)	C17—H17	0.9300
C4—C5	1.510 (4)	C18—H18	0.9300
C5—H5A	0.9600	C19—C20	1.380 (4)
C5—H5B	0.9600	C19—C24	1.391 (4)
C5—H5C	0.9600	C20—C21	1.383 (3)
C6—C7	1.381 (3)	C20—H20	0.9300
C6—H6	0.9300	C21—C22	1.370 (4)
C7—C8	1.385 (3)	C21—H21	0.9300
C7—H7	0.9300	C22—O1	1.373 (3)
C8—C9	1.488 (3)	C22—C23	1.373 (4)
C9—C10	1.366 (3)	C23—C24	1.381 (3)
C9—C14	1.430 (3)	C23—H23	0.9300
C10—C11	1.397 (3)	C24—H24	0.9300
C10—H10	0.9300	C25—O1	1.420 (4)
C11—C12	1.363 (4)	C25—H25A	0.9600
C11—H11	0.9300	C25—H25B	0.9600
C12—C13	1.430 (3)	C25—H25C	0.9600
C12—C19	1.488 (3)		
			
C2—C1—H1A	109.5	C18—C13—C12	121.9 (2)
C2—C1—H1B	109.5	C14—C13—C12	119.8 (2)
H1A—C1—H1B	109.5	C15—C14—C13	118.0 (2)
C2—C1—H1C	109.5	C15—C14—C9	121.8 (2)
H1A—C1—H1C	109.5	C13—C14—C9	120.2 (2)
H1B—C1—H1C	109.5	C16—C15—C14	121.4 (3)
C8—C2—C3	118.4 (2)	C16—C15—H15	119.3
C8—C2—C1	122.1 (2)	C14—C15—H15	119.3
C3—C2—C1	119.5 (3)	C15—C16—C17	120.4 (3)
C4—C3—C2	122.9 (3)	C15—C16—H16	119.8
C4—C3—H3	118.5	C17—C16—H16	119.8
C2—C3—H3	118.5	C18—C17—C16	120.1 (3)
C6—C4—C3	117.8 (3)	C18—C17—H17	120.0
C6—C4—C5	121.9 (3)	C16—C17—H17	120.0
C3—C4—C5	120.3 (3)	C17—C18—C13	121.6 (3)
C4—C5—H5A	109.5	C17—C18—H18	119.2
C4—C5—H5B	109.5	C13—C18—H18	119.2
H5A—C5—H5B	109.5	C20—C19—C24	117.0 (2)
C4—C5—H5C	109.5	C20—C19—C12	120.9 (2)
H5A—C5—H5C	109.5	C24—C19—C12	122.1 (2)
H5B—C5—H5C	109.5	C19—C20—C21	122.2 (3)
C4—C6—C7	120.7 (3)	C19—C20—H20	118.9
C4—C6—H6	119.7	C21—C20—H20	118.9
C7—C6—H6	119.7	C22—C21—C20	119.7 (3)
C6—C7—C8	122.1 (3)	C22—C21—H21	120.2
C6—C7—H7	119.0	C20—C21—H21	120.2
C8—C7—H7	119.0	C21—C22—O1	124.4 (3)
C7—C8—C2	118.1 (2)	C21—C22—C23	119.5 (3)
C7—C8—C9	118.8 (2)	O1—C22—C23	116.0 (3)
C2—C8—C9	123.1 (2)	C22—C23—C24	120.5 (3)
C10—C9—C14	117.6 (2)	C22—C23—H23	119.8
C10—C9—C8	120.0 (2)	C24—C23—H23	119.8
C14—C9—C8	122.3 (2)	C23—C24—C19	121.2 (3)
C9—C10—C11	122.3 (3)	C23—C24—H24	119.4
C9—C10—H10	118.9	C19—C24—H24	119.4
C11—C10—H10	118.9	O1—C25—H25A	109.5
C12—C11—C10	122.1 (3)	O1—C25—H25B	109.5
C12—C11—H11	119.0	H25A—C25—H25B	109.5
C10—C11—H11	119.0	O1—C25—H25C	109.5
C11—C12—C13	118.0 (2)	H25A—C25—H25C	109.5
C11—C12—C19	120.0 (2)	H25B—C25—H25C	109.5
C13—C12—C19	122.0 (2)	C22—O1—C25	117.3 (2)
C18—C13—C14	118.4 (2)		
			
C8—C2—C3—C4	0.2 (4)	C12—C13—C14—C9	2.4 (3)
C1—C2—C3—C4	−178.4 (3)	C10—C9—C14—C15	179.2 (2)
C2—C3—C4—C6	1.4 (4)	C8—C9—C14—C15	3.5 (4)
C2—C3—C4—C5	−178.2 (3)	C10—C9—C14—C13	0.0 (3)
C3—C4—C6—C7	−1.2 (4)	C8—C9—C14—C13	−175.7 (2)
C5—C4—C6—C7	178.4 (3)	C13—C14—C15—C16	−3.2 (4)
C4—C6—C7—C8	−0.7 (4)	C9—C14—C15—C16	177.6 (2)
C6—C7—C8—C2	2.3 (4)	C14—C15—C16—C17	−0.3 (4)
C6—C7—C8—C9	−178.6 (2)	C15—C16—C17—C18	2.4 (4)
C3—C2—C8—C7	−2.0 (4)	C16—C17—C18—C13	−0.9 (4)
C1—C2—C8—C7	176.6 (3)	C14—C13—C18—C17	−2.6 (4)
C3—C2—C8—C9	178.9 (2)	C12—C13—C18—C17	178.8 (3)
C1—C2—C8—C9	−2.5 (4)	C11—C12—C19—C20	−53.8 (3)
C7—C8—C9—C10	−63.5 (3)	C13—C12—C19—C20	126.4 (3)
C2—C8—C9—C10	115.5 (3)	C11—C12—C19—C24	123.9 (3)
C7—C8—C9—C14	112.1 (3)	C13—C12—C19—C24	−56.0 (3)
C2—C8—C9—C14	−68.9 (3)	C24—C19—C20—C21	0.5 (4)
C14—C9—C10—C11	−1.1 (4)	C12—C19—C20—C21	178.2 (2)
C8—C9—C10—C11	174.7 (2)	C19—C20—C21—C22	−0.3 (4)
C9—C10—C11—C12	−0.3 (4)	C20—C21—C22—O1	−179.9 (2)
C10—C11—C12—C13	2.7 (4)	C20—C21—C22—C23	−0.2 (4)
C10—C11—C12—C19	−177.2 (2)	C21—C22—C23—C24	0.6 (4)
C11—C12—C13—C18	174.9 (2)	O1—C22—C23—C24	−179.7 (2)
C19—C12—C13—C18	−5.2 (3)	C22—C23—C24—C19	−0.4 (4)
C11—C12—C13—C14	−3.7 (3)	C20—C19—C24—C23	−0.1 (4)
C19—C12—C13—C14	176.2 (2)	C12—C19—C24—C23	−177.8 (2)
C18—C13—C14—C15	4.5 (3)	C21—C22—O1—C25	−11.4 (4)
C12—C13—C14—C15	−176.8 (2)	C23—C22—O1—C25	168.9 (3)
C18—C13—C14—C9	−176.2 (2)		

### Hydrogen-bond geometry (Å, º)

Cg is the centroid of the C13–C18 ring.

**Table:**

*D*—H···*A*	*D*—H	H···*A*	*D*···*A*	*D*—H···*A*
C25—H25*C*···*Cg*^i^	0.96	2.78	3.597 (5)	144

Symmetry code: (i) *x*−1, *y*, *z*.
